# Interpretable machine learning model using CT body composition combined with inflammatory and nutritional indicators to predict pathological complete response after neoadjuvant therapy in breast cancer: a retrospective study

**DOI:** 10.7717/peerj.21051

**Published:** 2026-03-30

**Authors:** Linhua Zhong, Qiao Zeng, Fei Zou, Mingxian Gong, Lan Liu, Yongjie Zhou

**Affiliations:** Department of Radiology, Jiangxi Cancer Hospital & Institute, Jiangxi Clinical Research Center for Cancer, The Second Affiliated Hospital of Nanchang Medical College, Nanchang, Jiangxi, China

**Keywords:** Breast cancer, Neoadjuvant therapy, Machine learning, Body composition, Pathological complete response

## Abstract

**Objective:**

Accurate prediction of pathological complete response (pCR) following neoadjuvant therapy (NAT) is critical for optimizing treatment in breast cancer. This study develops and validates an interpretable, cost-effective machine learning (ML) model integrating computed tomography (CT)-based body composition parameters with routine inflammatory and nutritional biomarkers to predict pCR.

**Methods:**

In this retrospective single-center study (*n* = 189; January 2019–June 2023), patients were divided into training (*n* = 142) and independent temporal test (*n* = 47) sets. CT-based body composition parameters and blood test variables were analyzed. Independent predictors were identified *via* Least Absolute Shrinkage and Selection Operator and multivariate logistic regression. Eight ML algorithms were compared, and the optimal model was selected based on Area Under the Curve (AUC), calibration, and clinical utility. SHapley Additive exPlanations (SHAP) analysis visualized predictive contributions.

**Results:**

Six independent predictors were identified: visceral adipose tissue density, skeletal muscle density, intramuscular adipose tissue content, albumin-to-alkaline phosphatase ratio, systemic inflammation response index, and molecular subtype. The eXtreme Gradient Boosting (XGBoost) model demonstrated superior performance, achieving an area under the curve (AUC) of 0.888 (95% CI [0.837–0.939]) in internal validation and 0.831 (95% CI [0.723–0.938]) in the independent test set. The model exhibited good calibration (Brier score = 0.180). SHAP analysis highlighted the contribution of host-related factors alongside tumor biology.

**Conclusions:**

This interpretable ML model effectively integrates host-related body composition and inflammatory-nutritional markers to predict pCR. By utilizing routinely available data, this approach offers a practical, accessible tool for initial risk stratification, complementing existing imaging-based strategies and supporting personalized clinical decision-making.

## Introduction

Breast cancer remains one of the most prevalent malignancies worldwide and a leading cause of cancer-related mortality among women (Bray et al., 2024). With the continuous evolution of cancer treatment strategies, modern oncology has increasingly shifted toward personalized and risk-adapted therapeutic approaches (Sonkin, Thomas & Teicher, 2024). Neoadjuvant therapy (NAT) has become an integral component of breast cancer management, particularly for patients with stage II–III disease and selected high-risk early-stage subtypes, as it enables tumor downstaging, increases breast-conserving surgery rates, and provides an *in vivo* assessment of treatment response (Korde et al., 2021). Pathological complete response (pCR) after NAT is widely recognized as a clinically meaningful endpoint that correlates with favorable prognosis in specific breast cancer subtypes (Cortazar et al., 2014; Hirmas, Holtschmidt & Loibl, 2024). However, pCR rates vary across molecular subtypes and treatment regimens, and a substantial proportion of patients still fail to achieve pCR (Korde et al., 2021; Schmid et al., 2022; Romeo et al., 2021). Therefore, accurately predicting pCR before initiation of NAT is of substantial clinical importance, as it may guide treatment selection, optimize therapeutic intensity, and improve patient outcomes (Korde et al., 2021).

Recent research on pCR prediction has largely focused on imaging-based approaches, particularly dynamic contrast-enhanced magnetic resonance imaging (DCE-MRI), radiomics, and deep learning models derived from primary tumors, peritumoral tissues, and axillary lymph nodes (Sharafeldeen et al., 2025; Peng et al., 2022; Gamal et al., 2024; D’Anna et al., 2025; Iwase et al., 2021). Although these methods have shown promising predictive performance, they often rely on complex imaging pipelines (*e.g.*, standardized acquisition, segmentation, and feature extraction), which may limit clinical accessibility and generalizability (Traverso et al., 2018; Park et al., 2019; Zwanenburg et al., 2020).

Importantly, treatment response in breast cancer is influenced not only by tumor-intrinsic biology but also by host-related factors such as systemic inflammation, immune status, and nutritional condition (Feeney et al., 2024; Yamamoto, Kawada & Obama, 2021). In parallel, body composition—particularly skeletal muscle and adipose tissue characteristics—has emerged as a clinically relevant marker reflecting metabolic reserve and treatment resilience (Iwase et al., 2021). Computed tomography (CT), routinely performed for staging in many patients, provides an objective and reproducible means of assessing body composition without additional cost or patient burden (Pickhardt, Summers & Garrett, 2021; Zhou et al., 2025a; Zhou et al., 2025b). Machine learning (ML) offers a powerful framework for integrating multidimensional clinical data and capturing complex, non-linear relationships among predictors (Deo, 2015). However, for real-world clinical adoption, predictive models must not only demonstrate accuracy but also provide interpretability to support individualized decision-making.

Against this background, the present study aims to develop and validate an interpretable ML model that integrates CT-based body composition parameters with systemic inflammatory and nutritional indicators to predict pCR in breast cancer patients undergoing neoadjuvant therapy. By leveraging routinely available host-related features and explainable ML techniques, this work seeks to complement existing imaging-centered approaches and provide a clinically practical tool for personalized treatment planning.

## Materials and Methods

### General information

**Figure 1:**
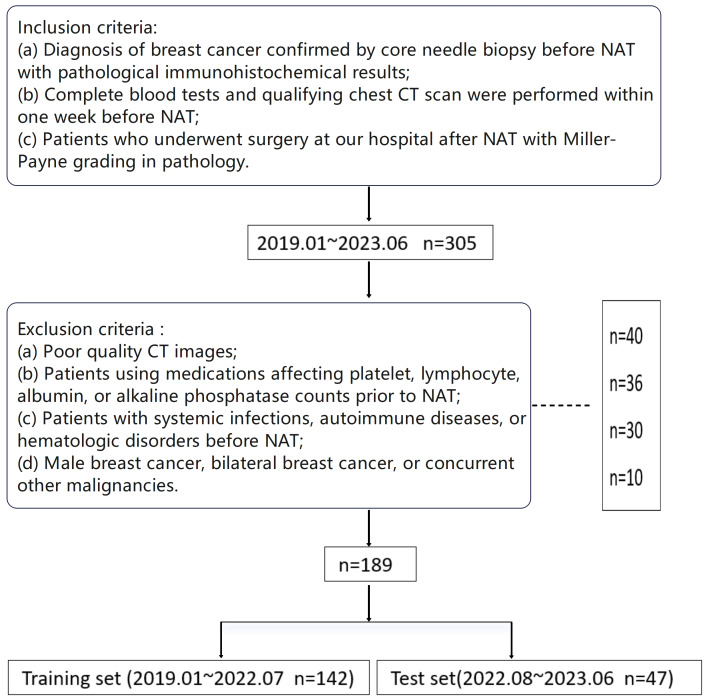
Flowchart of included BC patients; BC, breast cancer.

This retrospective, single-center study was approved by the Ethics Review Committee of Jiangxi Cancer Hospital (Approval No: 2023ky126), and informed consent was waived. Given its retrospective design, the cohort is subject to potential selection and information bias; to mitigate these risks, we consecutively enrolled eligible patients using prespecified criteria and applied a temporal split into training and independent test sets to approximate real-world generalization. Personally identifiable information was anonymized during data analysis. We consecutively collected data from breast cancer patients meeting the inclusion criteria at Jiangxi Cancer Hospital between January 2019 and June 2023. All enrolled patients completed NAT cycles in accordance with Chinese Society of Clinical Oncology Guidelines (Li et al., 2024). Inclusion criteria were: (a) diagnosis of breast cancer confirmed by core needle biopsy before NAT with pathological immunohistochemical results; (b) complete blood tests and qualifying chest CT scan were performed within one week before NAT; (c) patients who underwent surgery at our hospital after NAT with Miller-Payne grading in pathology. Exclusion criteria were: (a) poor quality CT images; (b) patients using medications affecting platelet, lymphocyte, albumin, or alkaline phosphatase counts prior to NAT; (c) patients with systemic infections, autoimmune diseases, or hematologic disorders before NAT; (d) male breast cancer, bilateral breast cancer, or concurrent other malignancies. Figure 1 shows the detailed patient selection process.

### Pathological examination

Before NAT, all patients underwent core needle biopsy; according to the American Society of Clinical Oncology (ASCO) and the College of American Pathologists (CAP) testing guidelines (Allison et al., 2020), if at least 1% of tumor cell nuclei are stained, the tumor is considered hormone receptor (HR) positive (Estrogen Receptor (ER)-positive or Progesterone Receptor (PR)-positive), with both ER and PR negative defined as HR-negative. Based on ASCO/CAP HER2 testing guidelines (Wolff et al., 2023), tumors with Immunohistochemistry (IHC) score 3+ or those scored 2+ with HER2 gene amplification confirmed by fluorescence *in situ* hybridization are considered HER2 positive. Ki67 proliferation index >20% indicates high expression, ≤20% indicates low expression. Tumors are classified into four subtypes based on HR and HER2 status: HR+HER2-, HR+HER2+, HR-HER2+, and HR-HER2-. Response to NAT was evaluated using Miller-Payne grading (Ogston et al., 2003), with pCR defined as the absence of residual invasive tumor cells in the primary breast lesion and no cancer metastasis in axillary lymph nodes post-surgery (ypT0/Tis ypN0).

### Clinical data collection and definition of variables

Clinical data include age, height, weight, the initial maximum tumor diameter (MTD), and body mass index (BMI), with BMI calculated as weight (kg) divided by height squared (m^2^). Pretreatment blood tests (within one week before NAT) provided white blood cell, neutrophil, monocyte, and lymphocyte counts, as well as albumin, globulin, bilirubin, and alkaline phosphatase levels. From these routine measures, we derived composite inflammatory and nutritional indices—including the neutrophil-to-lymphocyte ratio (NLR), derived NLR (dNLR), platelet-to-lymphocyte ratio (PLR), lymphocyte-to-monocyte ratio (LMR), systemic immune-inflammation index (SII), systemic inflammation response index (SIRI), albumin-to-alkaline phosphatase ratio (AAPR), albumin-to-globulin ratio (AGR), prognostic nutritional index (PNI), albumin lymphocyte index (ALI), lymphocyte–albumin product (LA), and lymphocyte–monocyte score (LMS)—using standard formulas (Table S1). These indices were selected because they are routinely available prior to NAT and capture complementary aspects of host immune–inflammatory status and nutritional reserve that are biologically relevant to treatment tolerance and response.

### CT scanning and body composition measurement

**Figure 2:**
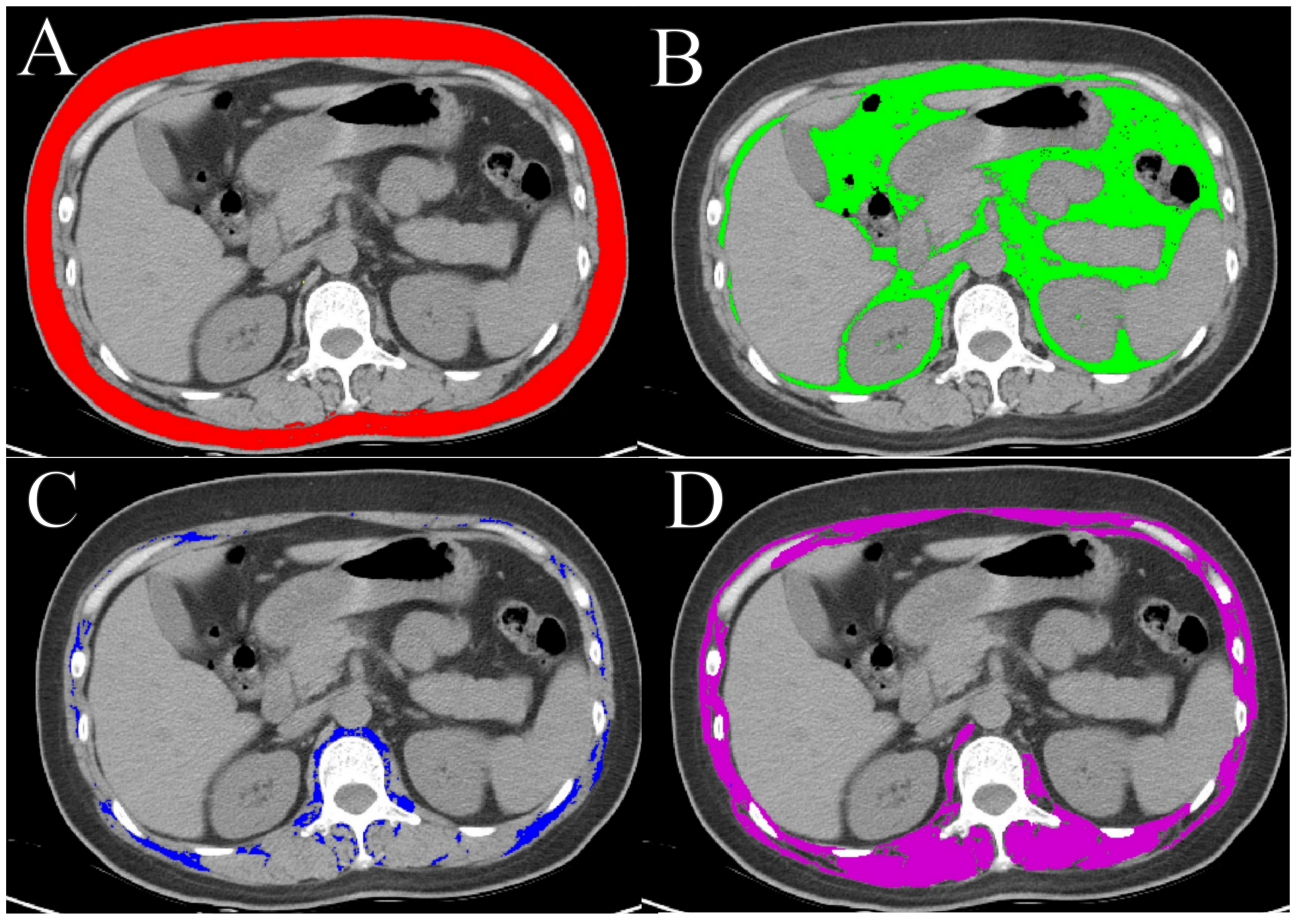
Body composition segmentation at the L1 level based on CT images. (A) Subcutaneous adipose tissue (SAT, red); (B) Visceral adipose tissue (VAT, green); (C) Intermuscular adipose tissue (IMAT, blue); (D) Skeletal muscle (SM, purple).

Within one week before NAT, all patients underwent unenhanced chest CT from the thoracic inlet to the adrenal glands. We quantified body composition at the L1 vertebral level because this slice was consistently available on routine pretreatment chest CT, enabling assessment without additional imaging or radiation. Prior studies suggest that L1-based muscle and adipose measures from chest CT can serve as practical surrogates for overall body composition; however, because body composition and CT protocols vary across populations and centers, the generalizability of L1-derived metrics should be interpreted cautiously and requires external validation. Details of the CT scanners and scanning settings are provided in Table S2. The images were accessed through the Picture Archiving and Communication System and analyzed using RadiAnt Viewer software. As an alternative to abdominal CT, a single CT image at the L1 vertebral level is a feasible method for assessing whole-body tissue quality (Zou et al., 2025); experienced radiologists identified the L1 level starting from the first thoracic vertebra, saving the cross-sectional DICOM images. Selected images were analyzed with semi-automated software Slicomatic 5.0, as shown in Fig. 2. The Hounsfield unit (HU) ranges for tissue components were defined as: subcutaneous adipose tissue (SAT) −190 to −30 HU; skeletal muscle (SM) −29 to +150 HU; intermuscular adipose tissue (IMAT) and visceral adipose tissue (VAT) −150 to −30 HU (Zou et al., 2025; Zhou et al., 2025a; Zhou et al., 2025b). L1-skeletal muscle (L1-SM) includes abdominal wall muscles, intercostal muscles, diaphragm, lumbar muscles, and paraspinal muscles (Zou et al., 2025). These regions were standardized by their height squared (m^2^), deriving SAT index (SATI), SM index (SMI), and IMAT index (IMATI). The mean HU value within each region was recorded to characterize tissue density, including SM density (SMD), VAT density (VATD), SAT density (SATD), and IMAT density (IMATD). Intramuscular adipose tissue content (IMAC) was evaluated by dividing the attenuation value of IMAT by that of SM (Zhou et al., 2025a; Zhou et al., 2025b; Zou et al., 2025). Total adipose tissue (TAT) area was estimated by summing SAT and VAT areas, with the VAT to SAT area ratio (VSR) calculated subsequently. Two radiologists with 9 years of experience independently assessed the body composition parameters in a blinded manner, and the mean of their measurements was used as final data.

### Variable selection and model building

Feature selection was conducted in the training set using a three-step strategy to balance interpretability and overfitting control, given the modest sample size. First, candidate variables were screened using univariable logistic regression with a liberal threshold (*P* < 0.10) to avoid prematurely excluding potentially relevant predictors. Second, LASSO regression with 10-fold cross-validation (*via* cv.glmnet) was applied to shrink coefficients, address multicollinearity, and select a parsimonious feature set; this cross-validation strategy ensured stable penalty parameter selection. Third, multivariable logistic regression was performed to identify independent predictors of pCR and report clinically interpretable effect-size estimates.

The detailed model building process is as follows:

(a) Data division: Patients from January 2019 to July 2022 (142 cases) form the training set; those from August 2022 to June 2023 (47 cases) constitute the independent test set.
(b) Comparison of multiple ML models: Based on the identified independent predictors, eight mainstream machine learning algorithms were constructed, including Extreme Gradient Boosting (XGBoost), Logistic Regression (LR), Random Forest (RF), Decision Tree (DT), Support Vector Machine (SVM), k-Nearest Neighbors (KNN), Gaussian Naive Bayes (GNB), and LightGBM. Model development was implemented in Python using the scikit-learn (0.22.1), lightgbm (3.2.1), and xgboost (1.2.1) libraries. We employed five-fold cross-validation to evaluate the training set, randomly partitioning the data into five subsets (80% for training and 20% for validation in each fold). Final model performance was assessed on the independent test set. The optimal model was identified based on the area under the receiver operating characteristic (ROC) curve (AUC) and calibration performance.
(c) Visualization of the optimal model: Shapley Additive Explanations (SHAP) was applied to visualize feature importance and rankings (Zhou et al., 2025a; Zhou et al., 2025b), as well as to explain individual predictions, aiding in understanding the model’s decisions and identifying potential biases.

### Statistical analysis

Cutoff values for body-composition parameters and blood biomarkers were derived only in the training set using ROC analysis with pCR as the endpoint, applying the Youden index (maximizing sensitivity + specificity) (Table S3). These prespecified cutoffs were then applied unchanged to the independent test set to prevent data leakage. Categorical variables are presented as frequencies (percentages) and compared using the chi-square test. Correlations among selected variables were assessed with Kendall’s tau. The 95% confidence intervals (CI) for the AUC were calculated using distinct approaches based on the validation step. For the independent test set, the 95% CI were estimated using a parametric normal approximation based on the standard error. For the internal 5-fold cross-validation, the 95% CI were derived from the standard error of the mean (SEM) of the AUC scores calculated across the five validation folds. The DeLong test was utilized specifically for evaluating the statistical significance of differences between the models’ ROC curves. Model calibration was evaluated using calibration plots, and clinical utility was assessed using decision curve analysis (DCA). Comparative model performance was assessed using the DeLong test, integrated discrimination improvement (IDI), and net reclassification improvement (NRI). All analyses were performed in Python (v3.5.6) and R (v3.6.3), with two-sided *P* < 0.05 considered statistically significant.

## Results

### Patient population characteristics

We retrospectively collected data from 305 breast cancer patients receiving NAT, of whom 189 were included after screening; among them, 71 patients (37.57%) were aged ≤45 years, and 118 patients (62.43%) were older than 45 years; 95 patients (50.27%) had a BMI < 24 kg/m^2^, and 94 patients (49.73%) had a BMI ≥24 kg/m^2^; 163 patients (86.24%) had an initial tumor MTD ≤5 cm, while 26 patients (13.76%) had MTD > 5 cm, with 100 patients (52.91%) achieving pCR after NAT, and 89 patients (47.09%) did not achieve pCR. The demographic and clinical-pathological features of patients in the training and test groups were well balanced (*P* > 0.05), as shown in Table 1.

**Table 1:** Baseline characteristics of included BC patients.

**Variables**	**Dataset, No (%)**				**Univariate analysis**	
	**Total sets (*n* = 189)**	**Training set (*n* = 142)**	**Test set (*n* = 47)**	p-value^*^	**Odds Ratio (95% CI)**	p-value^**^
MTD				0.474		
≤ 5 cm	163 (86.243)	121 (85.211)	42 (89.362)			
>5 cm	26 (13.757)	21 (14.789)	5 (10.638)		0.626 (0.242, 1.618)	0.333
Age				0.356		
≤ 45 y	71 (37.566)	56 (39.437)	15 (31.915)			
>45 y	118 (62.434)	86 (60.563)	32 (68.085)		0.870 (0.443, 1.706)	0.684
BMI				0.834		
<24 kg/m^2^	95 (50.265)	72 (50.704)	23 (48.936)			
≥ 24 kg/m^2^	94 (49.735)	70 (49.296)	24 (51.064)		0.842 (0.436, 1.628)	0.609
HR				0.362		
Negative	75 (39.683)	59 (41.549)	16 (34.043)			
Positive	114 (60.317)	83 (58.451)	31 (65.957)		0.354 (0.178, 0.707)	0.003
Her2				0.836		
Negative	107 (56.614)	81 (57.042)	26 (55.319)			
Positive	82 (43.386)	61 (42.958)	21 (44.681)		8.224 (3.842, 17.606)	<0.001
Ki67				0.869		
Low	50 (26.455)	38 (26.761)	12 (25.532)			
High	139 (73.545)	104 (73.239)	35 (74.468)		2.977 (1.337, 6.628)	0.008
Molecular subtype				0.3		
HR positive, HER2 negative	68 (35.979)	48 (33.803)	20 (42.553)			
HR positive, HER2 positive	46 (24.339)	35 (24.648)	11 (23.404)		12.779 (4.369, 37.376)	<0.001
HR negative, HER2 positive	38 (20.106)	27 (19.014)	11 (23.404)		25.771 (7.317, 90.775)	<0.001
HR negative, HER2 negative	37 (19.577)	32 (22.535)	5 (10.638)		5.168 (1.790, 14.924)	0.002
Pathological type				0.639		
Invasive adenocarcinoma	103 (54.497)	76 (53.521)	27 (57.447)			
Non-specific invasive adenocarcinoma	86 (45.503)	66 (46.479)	20 (42.553)		0.744 (0.383, 1.442)	0.381
SAT				0.13		
Low	34 (17.989)	29 (20.423)	5 (10.638)			
High	155 (82.011)	113 (79.577)	42 (89.362)		0.403 (0.172, 0.944)	0.036
VAT				0.967		
Low	92 (48.677)	69 (48.592)	23 (48.936)			
High	97 (51.323)	73 (51.408)	24 (51.064)		0.507 (0.260, 0.989)	0.046
IMAT				0.584		
Low	139 (73.545)	103 (72.535)	36 (76.596)			
High	50 (26.455)	39 (27.465)	11 (23.404)		1.853 (0.877, 3.912)	0.106
SM				0.376		
Low	98 (51.852)	71 (50.000)	27 (57.447)			
High	91 (48.148)	71 (50.000)	20 (42.553)		0.505 (0.259, 0.984)	0.045
SATD				0.232		
Low	173 (91.534)	128 (90.141)	45 (95.745)			
High	16 (8.466)	14 (9.859)	2 (4.255)		2.105 (0.668, 6.629)	0.203
VATD				0.056		
Low	141 (74.603)	101 (71.127)	40 (85.106)			
High	48 (25.397)	41 (28.873)	7 (14.894)		2.822 (1.323, 6.022)	0.007
IMATD				0.903		
Low	106 (56.085)	80 (56.338)	26 (55.319)			
High	83 (43.915)	62 (43.662)	21 (44.681)		0.581 (0.297, 1.138)	0.113
SMD				0.578		
Low	83 (43.915)	64 (45.070)	19 (40.426)			
High	106 (56.085)	78 (54.930)	28 (59.574)		1.917 (0.979, 3.752)	0.058
AAPR				0.072		
Low	42 (22.222)	36 (25.352)	6 (12.766)			
High	147 (77.778)	106 (74.648)	41 (87.234)		3.766 (1.616, 8.777)	0.002
AGR				0.629		
Low	115 (60.847)	85 (59.859)	30 (63.830)			
High	74 (39.153)	57 (40.141)	17 (36.170)		0.533 (0.269, 1.055)	0.071
NLR				0.024		
Low	78 (41.270)	52 (36.620)	26 (55.319)			
High	111 (58.730)	90 (63.380)	21 (44.681)		0.473 (0.236, 0.948)	0.035
dNLR				0.101		
Low	62 (32.804)	42 (29.577)	20 (42.553)			
High	127 (67.196)	100 (70.423)	27 (57.447)		0.446 (0.213, 0.933)	0.032
PLR				0.625		
Low	148 (78.307)	110 (77.465)	38 (80.851)			
High	41 (21.693)	32 (22.535)	9 (19.149)		2.153 (0.959, 4.834)	0.063
PNI				0.266		
Low	60 (31.746)	42 (29.577)	18 (38.298)			
High	129 (68.254)	100 (70.423)	29 (61.702)		0.589 (0.285, 1.220)	0.154
SII				0.019		
Low	55 (29.101)	35 (24.648)	20 (42.553)			
High	134 (70.899)	107 (75.352)	27 (57.447)		0.522 (0.240, 1.135)	0.101
LA				0.291		
Low	100 (52.910)	72 (50.704)	28 (59.574)			
High	89 (47.090)	70 (49.296)	19 (40.426)		0.598 (0.308, 1.163)	0.130
ALI				0.559		
Low	122 (64.550)	90 (63.380)	32 (68.085)			
High	67 (35.450)	52 (36.620)	15 (31.915)		0.793 (0.400, 1.574)	0.508
LMR				0.374		
Low	78 (41.270)	56 (39.437)	22 (46.809)			
High	111 (58.730)	86 (60.563)	25 (53.191)		1.397 (0.709, 2.750)	0.334
LMS				0.633		
Low	111 (58.730)	82 (57.746)	29 (61.702)			
High	78 (41.270)	60 (42.254)	18 (38.298)		1.031 (0.530, 2.009)	0.927
SIRI				0.474		
Low	96 (50.794)	70 (49.296)	26 (55.319)			
High	93 (49.206)	72 (50.704)	21 (44.681)		0.424 (0.216, 0.832)	0.013
SATI				0.514		
Low	76 (40.212)	59 (41.549)	17 (36.170)			
High	113 (59.788)	83 (58.451)	30 (63.830)		0.574 (0.293, 1.127)	0.107
VATI				0.901		
Low	91 (48.148)	68 (47.887)	23 (48.936)			
High	98 (51.852)	74 (52.113)	24 (51.064)		0.538 (0.276, 1.048)	0.069
IMATI				0.366		
Low	135 (71.429)	99 (69.718)	36 (76.596)			
High	54 (28.571)	43 (30.282)	11 (23.404)		1.809 (0.876, 3.733)	0.109
SMI				0.982		
Low	145 (76.720)	109 (76.761)	36 (76.596)			
High	44 (23.280)	33 (23.239)	11 (23.404)		0.318 (0.135, 0.746)	0.008
VSR				0.204		
Low	118 (62.434)	85 (59.859)	33 (70.213)			
High	71 (37.566)	57 (40.141)	14 (29.787)		0.417 (0.208, 0.833)	0.013
TAT				0.192		
Low	27 (14.286)	23 (16.197)	4 (8.511)			
High	162 (85.714)	119 (83.803)	43 (91.489)		0.340 (0.130, 0.886)	0.027
IMAC				0.956		
Low	120 (63.492)	90 (63.380)	30 (63.830)			
High	69 (36.508)	52 (36.620)	17 (36.170)		1.866 (0.935, 3.724)	0.077
pCR				0.703		
No	100 (52.910)	74 (52.113)	26 (55.319)			
Yes	89 (47.090)	68 (47.887)	21 (44.681)			

**Notes.**

BC breast cancer MTD maximum tumor diameter BMI body mass index HR hormone receptor Her2 huan epidermal growth factor receptor-2 SAT subcutaneous adipose tissue VAT visceral adipose tissue IMAT intermuscular adipose tissue SM skeletal muscle SATD subcutaneous adipose tissue density VATD visceral adipose tissue density IMATD intermuscular adipose tissue density SMD skeletal muscle density AAPR albumin-to-alkaline phosphatase ratio AGR albumin-to-globulin ratio NLR neutrophil-to-lymphocyte ratio PLR platelet-to-lymphocyte ratio PNI prognostic nutritional index SII systemic immune-inflammation index LA lymphocyte-albumin ALI advanced lung cancer inflammation index LMR lymphocyte-to-monocyte ratio LMS lymphocyte-to-monocyte score SIRI system inflammation response index SATI subcutaneous adipose tissue index VATI visceral adipose tissue index IMATI intermuscular adipose tissue index SMI skeletal muscle index VSR visceral-to-subcutaneous fat area ratio IMAC intramuscular adipose content pCR pathological complete response

* Differences in data distribution between different cohorts.

** Univariate logistic regression analysis.

### Variables associated with pCR in the training set

**Figure 3:**
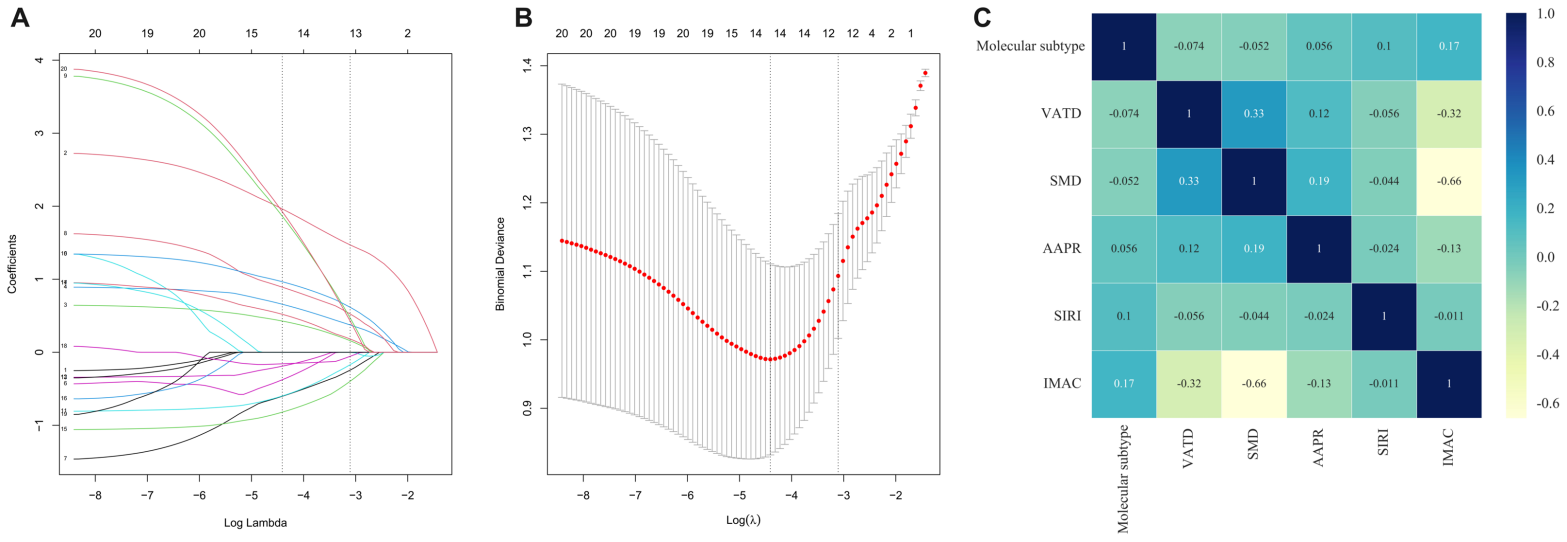
Selection of influencing factors using the LASSO regression model. (A) LASSO coefficient profiles of 17 predictive factors. (B) Determination of the optimal penalty parameter *λ* in the LASSO regression model. (C) Kendall’s correlation analysis among independent predictive factors.

Univariate LR analysis was performed on 35 indicators in the training cohort (Table 1). Seventeen indicators with *P*-values < 0.1 were included in LASSO regression analysis, with 10-fold cross-validation. Based on the minimum mean squared error at lambda = 0.012, 14 features with non-zero coefficients were selected: HER2, Ki67, Molecular subtype, SM, VATD, SMD, AAPR, AGR, PLR, SIRI, VSR, IMAC (Figs. 3A–3B). These variables were then incorporated into multivariate LR analysis, revealing that the independent predictors of pCR were: Molecular subtype with HR negative, HER2 negative (OR = 13.95; 95% CI [3.32–74.96]), VATD (OR = 4.21; 95% CI [1.16–19.20]), SMD (OR = 37.12; 95% CI [4.6–932.78]), AAPR (OR = 3.77; 95% CI [1.04–15.12]), SIRI (OR = 0.31; 95% CI [0.06–0.91]), and IMAC (OR = 37.78; 95% CI [4.37–1,003.41]) (Table 2). The correlation heatmap based on Kendall’s tau (Fig. 3C) showed that correlations among these six variables were all below 0.5, indicating no significant inter-variable correlations.

**Table 2:** Multivariate Cox regression analysis of independent predictors of pCR in the training set of BC.

**Variables**	**Training set (*n* = 142)**			***p-value***
	**Odds Ratio**	**95% CI**		
Molecular subtype				
HR positive, HER2 negative	Reference			
HR negative, HER2 negative	13.951	3.32, 74.957		0.001
VATD				
Low	Reference			
High	4.271	1.16, 19.204		0.039
SMD				
Low	Reference			
High	37.124	4.604, 932.777		0.005
AAPR				
Low	Reference			
High	3.774	1.041, 15.116		0.049
SIRI				
Low	Reference			
High	0.305	0.094, 0.905		0.037
IMAC				
Low	Reference			
High	37.781	4.371, 1,003.041		0.006

**Notes.**

BC breast cancer pCR pathological complete response HR hormone receptor Her2 huan epidermal growth factor receptor-2 VATD visceral adipose tissue density SMD skeletal muscle density AAPR albumin-to-alkaline phosphatase ratio SIRI system inflammation response index IMAC intramuscular adipose content

### Model development and evaluation

**Figure 4:**
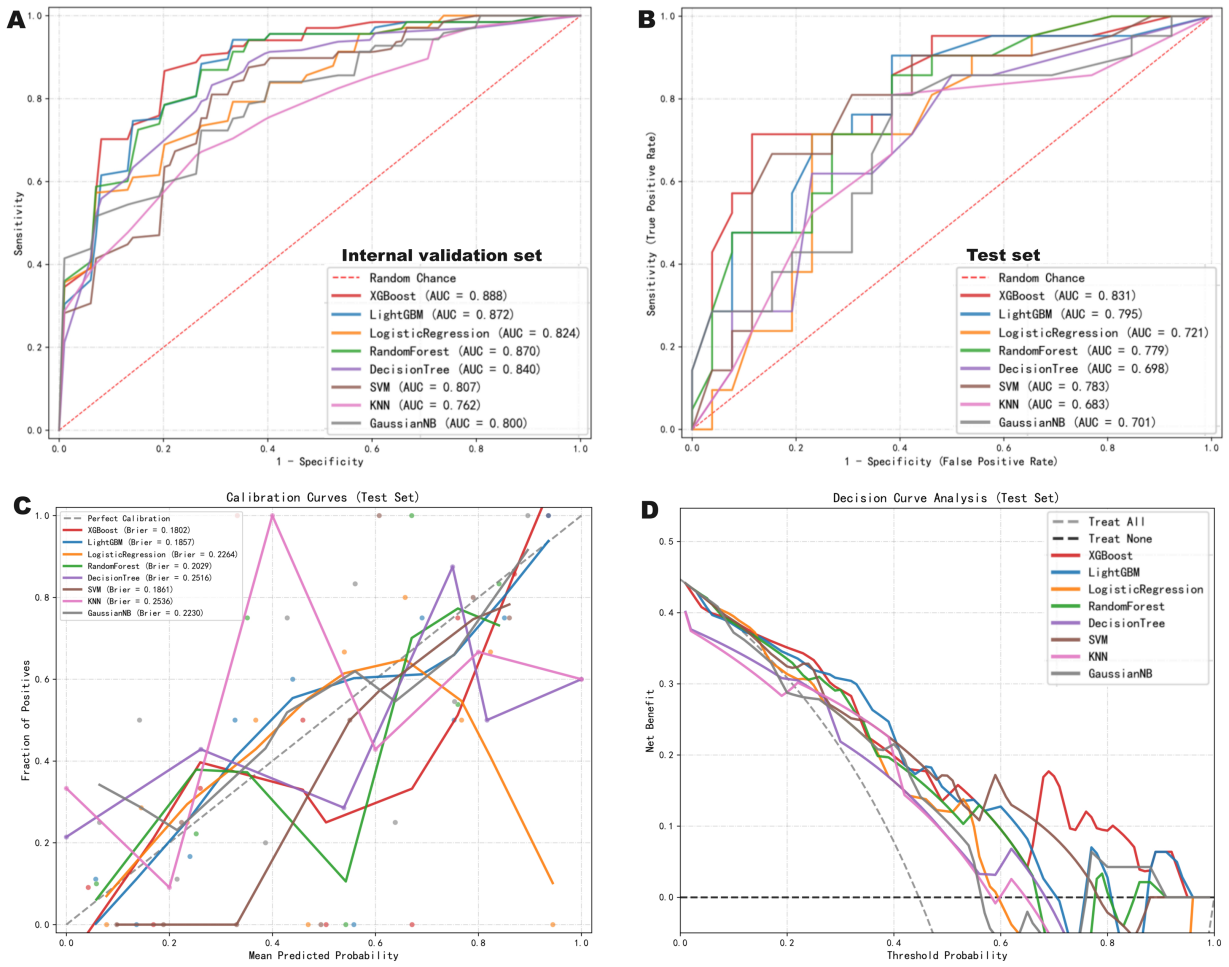
Performance evaluation of machine learning models for predicting pCR. (A) Receiver Operating Characteristic (ROC) curves of the eight machine learning models in the internal validation set. (B) ROC curves of the eight machine learning models in the independent test set. (C) Calibration curves of the models in the independent test set, with XGBoost demonstrating the best agreement between predicted and observed probabilities (lowest Brier score). (D) Decision Curve Analysis (DCA) in the independent test set, indicating that the XGBoost model provides superior net clinical benefit across a specific range of threshold probabilities compared to other models.

Table 3 summarizes the performance of the eight machine learning models in both the internal validation and independent test sets. The ROC curves for the internal validation and test cohorts are depicted in Figs. 4A and 4B, respectively. Notably, the XGBoost model achieved the highest AUC values in both the internal validation set (AUC = 0.888; 95% CI [0.837–0.939]) and the independent test set (AUC = 0.831; 95% CI [0.723–0.938]). Figures 4C and 4D illustrate the calibration curves and DCA for the independent test set. XGBoost demonstrated the best calibration accuracy with the lowest Brier score of 0.180. Furthermore, DCA demonstrated that XGBoost provided a higher net clinical benefit compared to other models across a specific range of threshold probabilities.

**Table 3:** Performance of multiple machine learning models in internal validation and test sets.

**Model**	**AUC (95% CI)**	**Accuracy**	**Sensitivity**	**Specificity**	**PPV**	**NPV**	**F1 score**
Internal validation set							
XGBoost	0.888 (0.837–0.939)	0.796	0.779	0.811	0.791	0.800	0.785
LightGBM	0.872 (0.809–0.935)	0.782	0.779	0.784	0.768	0.795	0.774
LogisticRegression	0.824 (0.789–0.859)	0.754	0.721	0.784	0.754	0.753	0.737
RandomForest	0.870 (0.819–0.921)	0.768	0.765	0.770	0.754	0.781	0.759
DecisionTree	0.840 (0.773–0.908)	0.761	0.838	0.689	0.713	0.823	0.770
SVM	0.807 (0.783–0.831)	0.711	0.677	0.743	0.708	0.714	0.692
KNN	0.762 (0.740–0.783)	0.711	0.662	0.757	0.714	0.709	0.687
GaussianNB	0.800 (0.765–0.834)	0.704	0.721	0.689	0.681	0.729	0.700
Test set							
XGBoost	0.831 (0.723–0.938)	0.681	0.714	0.654	0.625	0.739	0.667
LightGBM	0.795 (0.679–0.910)	0.702	0.714	0.692	0.652	0.750	0.682
LogisticRegression	0.721 (0.592–0.849)	0.681	0.714	0.654	0.625	0.739	0.667
RandomForest	0.779 (0.661–0.898)	0.681	0.714	0.654	0.625	0.739	0.667
DecisionTree	0.698 (0.567–0.829)	0.638	0.619	0.654	0.591	0.680	0.605
SVM	0.783 (0.665–0.901)	0.723	0.714	0.731	0.682	0.760	0.698
KNN	0.683 (0.550–0.816)	0.638	0.667	0.615	0.583	0.696	0.622
GaussianNB	0.701 (0.570–0.832)	0.660	0.667	0.654	0.609	0.708	0.636

**Notes.**

PPV positive predictive value NPV negative predictive value

**Figure 5:**
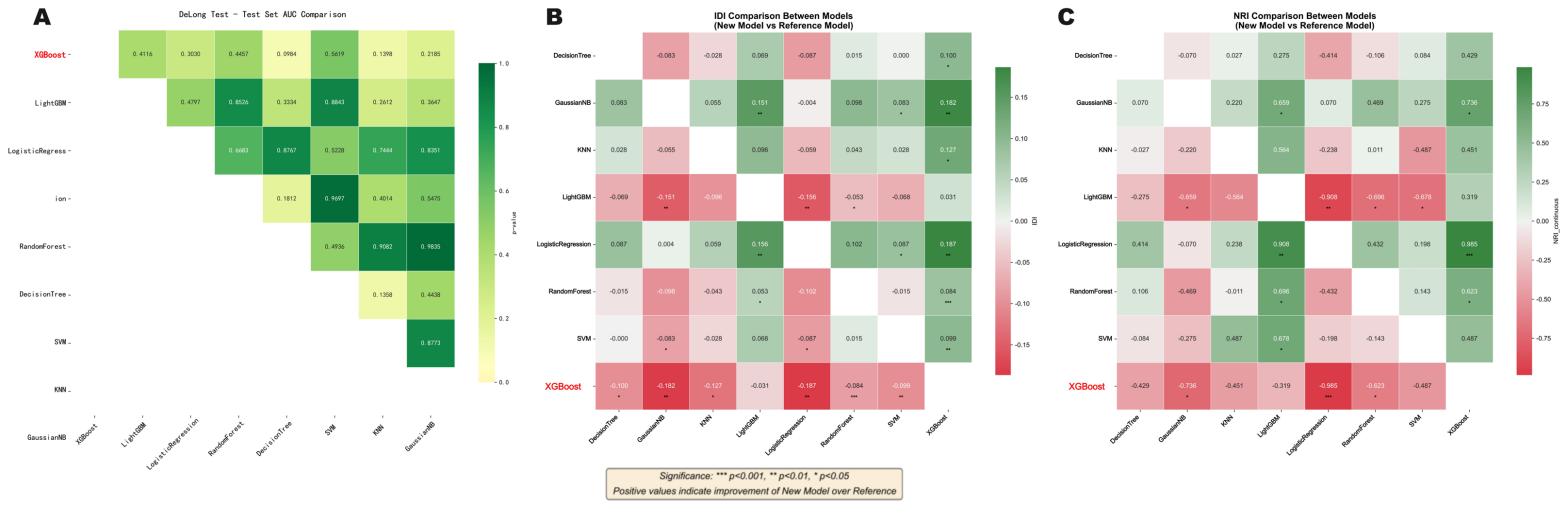
Statistical comparison and risk stratification improvement of the XGBoost model *versus* other algorithms. (A) DeLong test heatmap showing pairwise *P*-values for AUC comparisons among models in the independent test set. (B) Integrated Discrimination Improvement (IDI) analysis, quantifying the improvement in prediction probabilities offered by XGBoost compared to other models. (C) Net Reclassification Improvement (NRI) analysis, demonstrating the significant advantage of the XGBoost model in correctly reclassifying patients into appropriate risk categories.

As shown in the DeLong Heatmap (Fig. 5A), although XGBoost exhibited the highest absolute AUC in the test set, pairwise comparisons using the DeLong test revealed no statistically significant differences between XGBoost and other major models (all *p*-values > 0.05). However, in terms of prediction probability improvement metrics—specifically IDI and NRI—XGBoost demonstrated a significant advantage (Figs. 5B–5C), suggesting superior risk stratification capabilities. Given the limited size of the independent test set, these performance estimates should be interpreted cautiously and require confirmation in larger external cohorts.

### XGBoost model for SHAP

**Figure 6:**
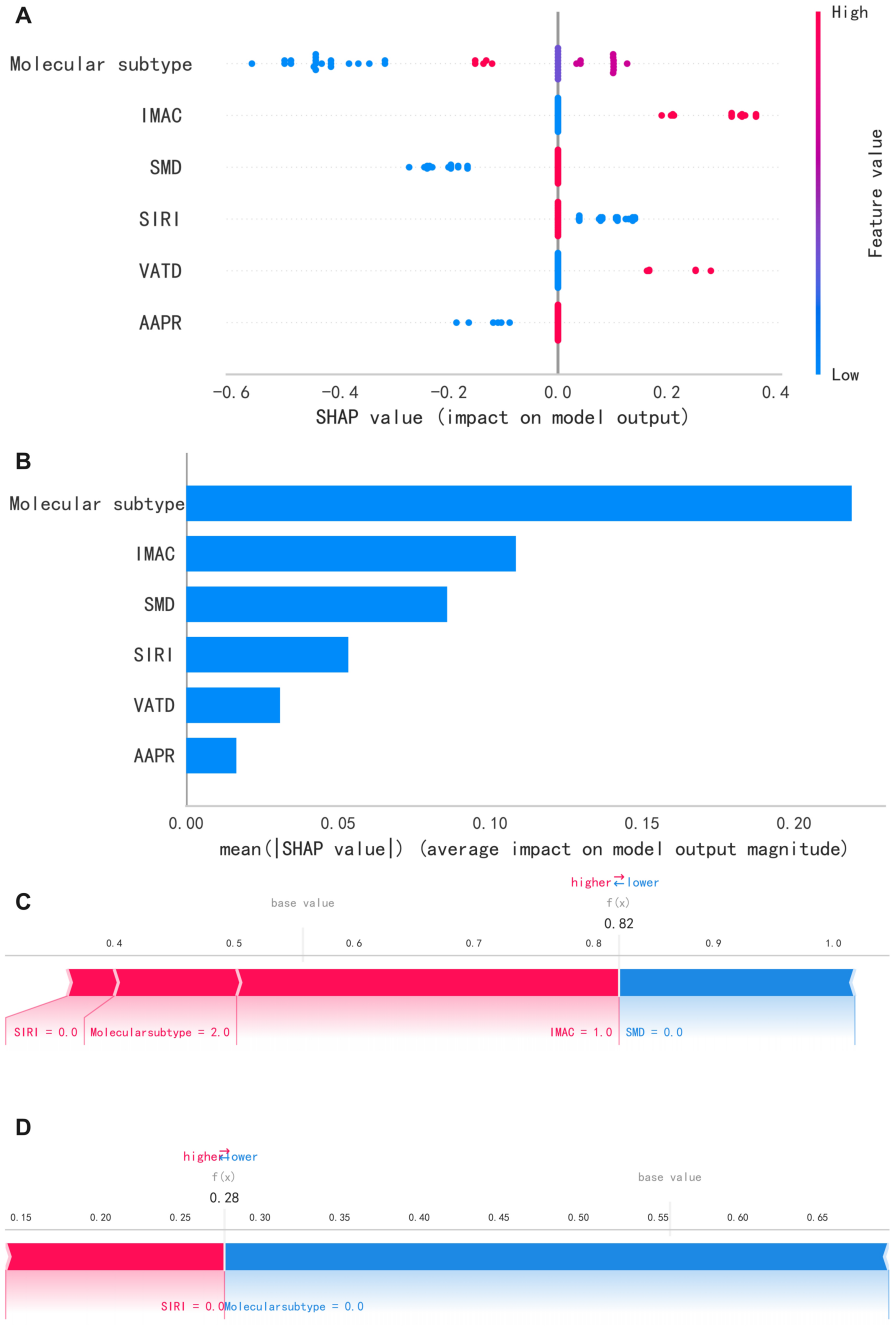
Summary analysis of SHAP values. The SHAP importance plot (A) shows the weight distribution of the six most important features in the model, while the variable contribution plot (B) visually presents the positive (red) or negative (blue) effects of each feature on the predicted probability. Panels (C) and (D) use SHAP to visualize individual prediction results for cases with pathological complete response (pCR) and non-pCR after neoadjuvant chemotherapy, respectively: the baseline value represents the model’s base prediction probability, and f(x) indicates the final predicted probability.

We computed global and local SHAP values for the XGBoost model to improve interpretability and potential clinical use. The SHAP summary plot (Fig. 6A) shows each feature’s contribution to the model’s predictions, and Fig. 6B ranks features by mean absolute SHAP values across the cohort. Two representative cases illustrate individual-level explanations: a patient with pCR (predicted probability 0.82; Fig. 6C) and a patient without pCR (0.28; Fig. 6D). SHAP values reflect model-based associations in this cohort and do not imply causality.

## Discussion

Histopathological examination of surgical specimens has long been considered the gold standard for evaluating the efficacy of NAT in breast cancer; however, its time delay limits early monitoring and dynamic assessment of tumor response. This study is the first to integrate CT-based body composition parameters with blood biomarkers, specifically identifying six critical independent predictors of pCR: VATD, SMD, IMAC, AAPR, SIRI, and molecular subtype. Using these features, we compared the performance of eight ML algorithms, where results demonstrate that the XGBoost model is an effective tool showing high accuracy and stability. Finally, SHAP analysis was employed to visualize the entire prediction process, providing interpretability for clinical decision-making.

Regarding body composition, VATD was identified as a significant independent predictor. In our cohort, elevated VATD was associated with a higher likelihood of achieving pCR. While the exact biological mechanism remains to be fully elucidated, visceral adipose tissue is metabolically active, secreting adipokines such as leptin and adiponectin that may modulate the tumor microenvironment. Previous studies, such as those by Iwase et al. (2016), have similarly observed that higher visceral fat measures can correlate with better treatment response in specific breast cancer populations, potentially reflecting a nutritional reserve that supports treatment tolerance, rather than a direct anti-tumor mechanism.

In parallel, skeletal muscle quality—assessed *via* SMD and IMAC—played a crucial role in our predictive model. Our findings indicate that higher SMD and lower IMAC (reflecting reduced myosteatosis) are predictive of pCR. Low muscle density and high intramuscular fat content are hallmarks of sarcopenia and systemic depletion. This state often implies compromised metabolic function, which can affect drug pharmacokinetics and distribution. Consistent with Lee et al. (2021), who demonstrated that skeletal muscle depletion correlates with lower pCR rates, our data suggests that maintaining muscle quality may be a relevant factor in supporting neoadjuvant treatment efficacy, though these associations should be interpreted as prognostic rather than strictly causal.

Systemic inflammatory and nutritional balance, captured by AAPR and SIRI, also contributed independently to the model. We observed that higher AAPR and lower SIRI levels were associated with improved responses. AAPR integrates albumin (a marker of nutritional status) and alkaline phosphatase, while SIRI reflects the balance of pro-inflammatory cells (neutrophils, monocytes) *versus* immune-regulatory lymphocytes. Furthermore, it is crucial to recognize that these systemic inflammation markers do not exist in isolation but may be modulated by broader perioperative and treatment-related factors. Recent evidence, such as the meta-analysis on the potential effect of general anesthetics in cancer surgery (Li et al., 2023), highlights that anesthetic management and surgical stress can significantly influence inflammatory cytokines and potential metastatic risks. Situating our findings within this wider oncologic context suggests that the inflammation signals observed here might be part of a dynamic host response that could be optimized through comprehensive peri-treatment management.

Molecular subtype remains a fundamental driver of therapeutic response. Consistent with extensive literature (Liu et al., 2024; Shi et al., 2023), our analysis confirmed that Triple-Negative Breast Cancer status (HR-negative/HER2-negative) is a strong predictor of pCR compared to luminal subtypes. This association is likely driven by the higher proliferation rates and increased chemosensitivity characteristic of this aggressive phenotype, reinforcing the necessity of including tumor biology alongside host features in predictive modeling.

A key distinction of our study is the prioritization of clinical accessibility over complexity. We acknowledge that numerous recent studies utilizing advanced imaging modalities, such as DCE-MRI and deep learning radiomics, have reported predictive performance that occasionally exceeds that of our model (Peng et al., 2022; Gamal et al., 2024; D’Anna et al., 2025; Iwase et al., 2021). However, these approaches often require specialized acquisition protocols, complex segmentation pipelines, and significant computational resources, which may limit their generalizability and utility in resource-constrained settings. In contrast, our system offers specific advantages: it relies exclusively on routine non-contrast CT scans and standard blood tests, incurring no additional cost, radiation exposure, or logistical burden. This makes our approach particularly appropriate as a widely deployable screening tool for initial risk stratification in centers where advanced MRI-based AI analysis is not yet feasible.

To integrate these multidimensional features, XGBoost was selected as the optimal algorithm. As a gradient boosting decision tree method, XGBoost offers distinct advantages in handling structured clinical data and capturing non-linear interactions between host and tumor factors (Zhou et al., 2025a; Zhou et al., 2025b). Its regularization strategies effectively reduce overfitting, while the integration of SHAP values addresses the “black box” nature of ML models, providing clinicians with interpretable, individualized risk profiles.

Several limitations of this study should be acknowledged. First, as a single-center retrospective analysis, the potential for selection bias exists, and our findings require validation in larger, multi-center cohorts. Second, our model relies on pre-NAT baseline measurements; future research should incorporate longitudinal data to assess how dynamic changes in body composition and inflammation during therapy influence outcomes. Lastly, while we adjusted for key clinical variables, unmeasured confounders—including specific perioperative management details discussed above—could influence the observed association.

## Conclusions

We developed an interpretable ML-based comprehensive model incorporating clinical-pathological features, body composition, and inflammation-nutrition indices to assess pCR after breast cancer NAT. By rigorously evaluating host-related factors alongside tumor biology, this study provides a practical, cost-effective tool that complements existing imaging-based strategies, supporting more personalized clinical decision-making.

##  Supplemental Information

10.7717/peerj.21051/supp-1 Supplemental Information 1 Abbreviations

10.7717/peerj.21051/supp-2 Supplemental Information 2 Supplementary Material

10.7717/peerj.21051/supp-3 Supplemental Information 3 The raw measurements

10.7717/peerj.21051/supp-4 Supplemental Information 4 Machine learning code

10.7717/peerj.21051/supp-5 Supplemental Information 5 Codebook In the raw data, the numerical codes of categorical variables were converted to their respective factors.

10.7717/peerj.21051/supp-6 Supplemental Information 6 STROBE checklist

## Additional Information and Declarations

### Data Availability

The following information was supplied regarding data availability: The raw data and code are available in the Supplemental Files.
