# Supplementary material for: Interpretable machine learning model using CT body composition combined with inflammatory and nutritional indicators to predict pathological complete response after neoadjuvant therapy in breast cancer: a retrospective study
Source: PeerJ. 2026 Mar 30;14:e21051. doi: 10.7717/peerj.21051 (PMC13045840; doi:10.7717/peerj.21051)
Supplement: Supplemental Information 2 [file peerj-14-21051-s002.docx]

**Supplementary Material**

1. ***Clinical parameters and body composition parameters***

| **Table S1. Clinical Parameters and Body Composition Parameters** | | | |
| --- | --- | --- | --- |
|  | **Parameter** | **Equation** | **Description** |
| Clinical Parameters | Body Mass Index (BMI) | BMI = Weight (kg) / Height² (m²) |  |
|  | Albumin-Alkaline Phosphatase Ratio (AAPR) | AAPR = Albumin (g/L) / ALP (IU/L) | Ratio calculated from albumin concentration and alkaline phosphatase activity |
|  | Albumin-Globulin Ratio (AGR) | AGR = Albumin Concentration (g/L) / Globulin Concentration (g/L) | Ratio calculated from albumin concentration and globulin concentration |
|  | Neutrophil-to-Lymphocyte Ratio (NLR) | NLR = Neutrophil Count / Lymphocyte Count | Ratio of neutrophil count to lymphocyte count, reflecting inflammatory status |
|  | Derived Neutrophil-to-Lymphocyte Ratio (dNLR) | dNLR = Neutrophil Count / (White Blood Cell Count - Neutrophil Count) | Ratio of neutrophil count to non-neutrophil count (white blood cell count minus neutrophil count) |
|  | Prognostic Nutritional Index (PNI) | PNI = Albumin (g/L) + 5 × Lymphocyte Count | Index combining albumin concentration and lymphocyte count, evaluating patients' nutritional status and prognosis |
|  | Platelet-to-Lymphocyte Ratio (PLR) | PLR = Platelet Count / Lymphocyte Count | Ratio of platelet count to lymphocyte count, reflecting inflammatory and coagulation status |
|  | Systemic Immune-Inflammation Index (SII) | SII = (Platelet Count × Neutrophil Count) / Lymphocyte Count | Index combining platelet, neutrophil and lymphocyte counts, evaluating systemic immune-inflammatory status |
|  | Albumin Lymphocyte Index (ALI) | ALI = BMI × Albumin / NLR | Comprehensive index calculated from BMI, albumin concentration and NLR |
|  | Lymphocyte-Albumin (LA) | LA = Lymphocyte Count × Albumin Concentration (g/dL) | Product of lymphocyte count and albumin concentration (unit: g/dL) |
|  | Lymphocyte-to-Monocyte Ratio (LMR) | LMR = Lymphocyte Count / Monocyte Count | Ratio of lymphocyte count to monocyte count |
|  | Lymphocyte-Monocyte Score (LMS) | 0 points: Lymphocyte ≥ 1.5×10⁹/L and Monocyte ≤ 0.80×10⁹/L; | Scoring index based on threshold values of lymphocyte and monocyte counts |
|  |  | 2 points: Lymphocyte < 1.5×10⁹/L and Monocyte > 0.80×10⁹/L; |  |
|  |  | 1 points: Other conditions |  |
|  | Systemic Inflammation Response Index (SIRI) | SIRI = (Monocyte Count × Neutrophil Count) / Lymphocyte Count | Index calculated from monocyte, neutrophil and lymphocyte counts, evaluating the degree of systemic inflammatory response |
| Body Composition Parameters | Subcutaneous Adipose Tissue(SAT) | HU Range: -190 to -30 HU | HU value range standard for defining subcutaneous fat tissue |
|  | Skeletal Muscle（SM） | HU Range: -29 to +150 HU | HU value range standard for defining skeletal muscle tissue |
|  | Intermuscular Adipose Tissue(IMAT) | HU Range: -150 to -30 HU | HU value range standard for defining intermuscular fat tissue |
|  | Visceral Adipose Tissue(VAT) | HU Range: -150 to -30 HU | HU value range standard for defining visceral fat tissue |
|  | Subcutaneous Adipose Tissue Index (SATI) | Derived by standardizing the area of subcutaneous by height squared (m²) | Standardized index reflecting subcutaneous adipose tissue, adjusted for height |
|  | Skeletal Muscle Index (SMI) | Derived by standardizing the area of skeletal muscle region by height squared (m²) | Standardized index reflecting skeletal muscle amount, adjusted for height |
|  | Intermuscular Adipose Tissue Index (IMATI) | Derived by standardizing the area of intermuscular adipose tissue region by height squared (m²) | Standardized index reflecting intermuscular adipose tissue amount, adjusted for height |
|  | Intramuscular Adipose Tissue Content (IMAC) | Calculation Formula: IMAC = Attenuation Value of IMAT / Attenuation Value of SM | Parameter evaluating intramuscular adipose tissue content, derived from the ratio of IMAT and SM attenuation values |
|  | Total Adipose Tissue (TAT) Area | Calculation Formula: TAT Area = SAT Area + VAT Area | Total area of adipose tissue, estimated by summing subcutaneous and visceral adipose tissue areas |
|  | VAT to SAT Area Ratio (VSR) | Calculation Formula: VSR = VAT Area / SAT Area (calculated subsequent to TAT area estimation) | Ratio of visceral adipose tissue area to subcutaneous adipose tissue area, reflecting the distribution of adipose tissue |

1. ***CT Scan methodology***

All patients with breast cancer underwent non-contrast chest CT scans within two weeks prior to diagnosis, covering the area from the thoracic inlet to the level of both adrenal glands. Detailed information regarding the scanning and reconstruction settings of the two CT scanners can be found in **Table S2**.

**Table1 S2. CT scanning protocol for patients with breast cancer.**

| **Manufacture** | **SIEMENS** | **GE** |
| --- | --- | --- |
| CT scanner | CT64 | CT64 |
| Scanner mode | SOMATOM Defifinition Edge | Revolution Frontier |
| Tube voltage (kV) | 120 | 120 |
| Tube current (mA) | 400 | 300 |
| Rotation time (s) | 0.5 | 0.5 |
| Collimation (mm) | 64*0.6 | 64*0.625 |
| Slice thickness (mm) | 1 | 1.25 |
| Matrix | 512 × 512 | 512 × 512 |
| Field of view (mm) | 350 | 350 |
| Kernel | B30f | standard |

CT, computed tomography.

1. ***Cut-off values for body composition and inflammatory nutritional index***

In the training set, ROC curves were utilized to determine cutoff values for various body composition parameters and blood indices, using pCR as the endpoint variable, to classify patients into different risk groups (**Table S3**), and these cutoff values were applied to the test set.

| **Table S3. Cut-off values for body composition and blood Parameters** | |
| --- | --- |
| **Variable** | **Cut-off values** |
| SAT | 64.17 |
| VAT | 61.41 |
| IMAT | 12.10 |
| SM | 79.77 |
| SATD | -91.28 |
| VATD | -76.95 |
| IMATD | -51.86 |
| SMD | 31.80 |
| AAPR | 0.49 |
| AGR | 1.53 |
| ALBI | -3.03 |
| NLR | 2.00 |
| dNLR | 1.50 |
| PLR | 189.74 |
| PNI | 49.50 |
| SII | 400.05 |
| LA | 72.72 |
| ALI | 552.08 |
| LMR | 4.96 |
| SIRI | 0.70 |
| SATI | 35.94 |
| VATI | 25.53 |
| IMATI | 5.00 |
| SMI | 36.41 |
| VSR | 0.76 |

Hu, Hounsfield Unit; SAT, subcutaneous adipose tissue; VAT, visceral adipose tissue; SM, skeletal Muscle; IMAT, intermuscular adipose tissue; SATD, subcutaneous adipose tissue density; VATD, visceral adipose tissue density; SMD, skeletal muscle density; IMATD, intermuscular adipose tissue density; IMAC, intramuscular adipose content; AGR, albumin-to-globulin Ratio; AAPR, albumin-to-alkaline phosphatase ratio; dNLR, derived neutrophil-to-lymphocyte ratio; PLR, platelet-to-lymphocyte ratio; PNI, prognostic nutritional index; SII, systemic immune-inflammation Index; SATI, subcutaneous adipose tissue index; VATI, visceral adipose tissue index; SMI, skeletal muscle index; IMATI, intermuscular adipose tissue index; VSR, visceral adipose tissue to subcutaneous adipose tissue area ratio.
