# Supplementary material for: Interpretable machine learning model using CT body composition combined with inflammatory and nutritional indicators to predict pathological complete response after neoadjuvant therapy in breast cancer: a retrospective study
Source: PeerJ. 2026 Mar 30;14:e21051. doi: 10.7717/peerj.21051 (PMC13045840; doi:10.7717/peerj.21051)
Supplement: Supplemental Information 6 [file peerj-14-21051-s006.doc]

STROBE Statement—Checklist of items that should be included in reports of ***cohort studies***

|  | Item No | Recommendation | Reported on Page  Number/Line  Number |
| --- | --- | --- | --- |
| **Title and abstract** | 1 | (*a*) Indicate the study’s design with a commonly used term in the title or the abstract | Page 1-2, Line 17-28 |
|  |  | (*b*) Provide in the abstract an informative and balanced summary of what was done and what was found | Page 2, Line 29-37 |
| Introduction | | |  |
| Background/rationale | 2 | Explain the scientific background and rationale for the investigation being reported | Page 2-4, Line 40-78 |
| Objectives | 3 | State specific objectives, including any prespecified hypotheses | Page 4, Line 79-81 |
| Methods | | |  |
| Study design | 4 | Present key elements of study design early in the paper | Page 4, Line 84-86 |
| Setting | 5 | Describe the setting, locations, and relevant dates, including periods of recruitment, exposure, follow-up, and data collection | Page 4, Line 86-89 |
| Participants | 6 | (*a*) Give the eligibility criteria, and the sources and methods of selection of participants. Describe methods of follow-up | Page 4-5, Line 89-97 |
|  |  | (*b*) For matched studies, give matching criteria and number of exposed and unexposed | Figure 1 |
| Variables | 7 | Clearly define all outcomes, exposures, predictors, potential confounders, and effect modifiers. Give diagnostic criteria, if applicable | Page 5-7, Line 99-143 |
| Data sources/ measurement | 8* | For each variable of interest, give sources of data and details of methods of assessment (measurement). Describe comparability of assessment methods if there is more than one group | Page 7, Line 144-146 |
| Bias | 9 | Describe any efforts to address potential sources of bias | Page 8, Line 160-163 |
| Study size | 10 | Explain how the study size was arrived at | The sample size adhered to the 10 Events Per Variable (EPV) principle, ensuring a minimum of 10 outcome events per candidate predictor variable for robust model stability. |
| Quantitative variables | 11 | Explain how quantitative variables were handled in the analyses. If applicable, describe which groupings were chosen and why | Page 8, Line 172-174 (Table S2) |
| Statistical methods | 12 | (*a*) Describe all statistical methods, including those used to control for confounding | Page 8, Line 174-179 |
|  |  | (*b*) Describe any methods used to examine subgroups and interactions | Page 8, Line 164-170 |
|  |  | (*c*) Explain how missing data were addressed | The patient characteristics are complete. |
|  |  | (*d*) If applicable, explain how loss to follow-up was addressed | Not Applicable |
|  |  | (*e*) Describe any sensitivity analyses | Page 4, Line 86 |
| Results | | |  |
| Participants | 13* | (a) Report numbers of individuals at each stage of study—eg numbers potentially eligible, examined for eligibility, confirmed eligible, included in the study, completing follow-up, and analysed | Page 9, Line 183-189 |
|  |  | (b) Give reasons for non-participation at each stage | Page 9, Line 183-189 |
|  |  | (c) Consider use of a flow diagram | Figure 1 |
| Descriptive data | 14* | (a) Give characteristics of study participants (eg demographic, clinical, social) and information on exposures and potential confounders | Table 1 |
|  |  | (b) Indicate number of participants with missing data for each variable of interest | Figure 1 |
|  |  | (c) Summarise follow-up time (eg, average and total amount) | Not Applicable |
| Outcome data | 15* | Report numbers of outcome events or summary measures over time | Not Applicable |
| Main results | 16 | (*a*) Give unadjusted estimates and, if applicable, confounder-adjusted estimates and their precision (eg, 95% confidence interval). Make clear which confounders were adjusted for and why they were included | Table 2 |
|  |  | (*b*) Report category boundaries when continuous variables were categorized | Table S2 |
|  |  | (*c*) If relevant, consider translating estimates of relative risk into absolute risk for a meaningful time period | Page 9, Line 191-200 |
| Other analyses | 17 | Report other analyses done—eg analyses of subgroups and interactions, and sensitivity analyses | Page 10, Line 212-221 |
| Discussion | | |  |
| Key results | 18 | Summarise key results with reference to study objectives | Page 11, Line 223-229 |
| Limitations | 19 | Discuss limitations of the study, taking into account sources of potential bias or imprecision. Discuss both direction and magnitude of any potential bias | Page 14, Line 303-310 |
| Interpretation | 20 | Give a cautious overall interpretation of results considering objectives, limitations, multiplicity of analyses, results from similar studies, and other relevant evidence | Page 14, Line 303-310 |
| Generalisability | 21 | Discuss the generalisability (external validity) of the study results | Page 15, Line 312-316 |
| Other information | | |  |
| Funding | 22 | Give the source of funding and the role of the funders for the present study and, if applicable, for the original study on which the present article is based | Page 15, Line 321-324 |

*Give information separately for exposed and unexposed groups.

**Note:** An Explanation and Elaboration article discusses each checklist item and gives methodological background and published examples of transparent reporting. The STROBE checklist is best used in conjunction with this article (freely available on the Web sites of PLoS Medicine at http://www.plosmedicine.org/, Annals of Internal Medicine at http://www.annals.org/, and Epidemiology at http://www.epidem.com/). Information on the STROBE Initiative is available at http://www.strobe-statement.org.
